# 

**Published:** 1877-09

**Authors:** 


*CONTENTS.*

| | Page |
|---|---|
| The Little Courtesies of Life | 239 |
| Coughing in Consumption | 241 |
| Influence of Christianity on Medical Science | 242 |
| Ignorance and Ill Health | 243 |
| Rage and Ruin | 243 |
| Kindness the Best Punishment | 244 |
| Grass in Rum | 245 |
| Valuable Table | 246 |
| Incurable Insanity | 246 |
| Consumption—A Suggestion | 247 |
| The Spirit Rapper | 248 |
| Premium on Babies | 251 |
| Our Proverbs | 253 |
| Wrecked Clergymen | 254 |
| Marrying Well | 255 |
| The Lifting Cure | 258 |
| Sea Sickness | 259 |
| Face Painting | 261 |
| A Filthy Atmosphere | 262 |
| The Latest Crazy Man | 262 |
| A Suggestion | 263 |
| The Erie Railway | 264 |
| Sick Children | 264 |

